# Chronic Low-Dose Phoxim Exposure Impairs Silk Production in *Bombyx mori* L. (Lepidoptera: Bombycidae) by Disrupting Juvenile Hormone Signaling-Mediated Fibroin Synthesis

**DOI:** 10.3390/toxics13060427

**Published:** 2025-05-23

**Authors:** Xinyi Xie, Jiayin Hou, Meng Li, Zhiyu Liu, Mengai He, Chenxi Li, Xiaohua Du, Liezhong Chen

**Affiliations:** 1 College of Chemical Engineering, Zhejiang University of Technology, Hangzhou 310014, China; 2 State Key Laboratory for Quality and Safety of Agro-Products, Zhejiang Academy of Agricultural Sciences, Hangzhou 310021, China

**Keywords:** silkworm, phoxim, chronic toxicity, silk gland, juvenile hormone

## Abstract

Phoxim is a pesticide extensively applied in mulberry fields, and residues may persist on leaves even after the recommended pre-harvest interval. However, the potential risks of these residues to *Bombyx mori* L. (Lepidoptera: Bombycidae) have long been overlooked. The results demonstrated that chronic low-dose exposure from the second to fifth instars significantly impaired silkworm development and silk production. Specifically, larvae in the 0.316 μg/mL treatment group (1/2 LC_50_) exhibited a significant reduction in body weight, while the cocoon shell ratio was significantly decreased in both the 0.079 μg/mL (1/8 LC_50_) and 1/2 LC_50_ groups. Cocoon deformities were observed in the 0.032 μg/mL (1/20 LC_50_), 1/8 LC_50_, and 1/2 LC_50_ groups. Histopathological analysis revealed silk gland damage in the treatment groups, with severity increasing with higher phoxim concentrations. Biochemical analyses indicated elevated malondialdehyde (MDA) levels accompanied by increased superoxide dismutase (SOD) and peroxidase (POD) activities. Notably, phoxim exposure selectively reduced juvenile hormone (JH) titers without affecting ecdysone titers. JH-regulated genes including the receptors *Met1* and *Met2*, and transcription factors *Kr-h1* and *Dimm* were downregulated, accompanied by suppressed expression of the fibroin synthesis gene *Fib-H*. These results collectively indicate that chronic low-concentration phoxim exposure disrupts endocrine regulation, damages silk gland integrity, and ultimately reduces silk production in silkworm.

## 1. Introduction

*Bombyx mori* L. (Lepidoptera: Bombycidae) is an economically important insect with a domestication history of over 5000 years in China, where it contributes to more than 80% of the global raw silk production [1,2,3]. However, the extensive use of chemical pesticides in agriculture poses severe challenges to the sericulture industry [4,5]. Reports indicate that pesticide-related silkworm poisoning events are leading to an annual decrease of over 30% in raw silk production in China, resulting in direct economic losses exceeding CNY 50 million [6,7].

Phoxim (O,O-diethyl-O-α-oximinophenyl cyanophosphorothioate) is an organophosphorus pesticide that is widely used for its high effectiveness. It exhibits both contact and oral toxicity and has a broad insecticidal spectrum, effectively targeting pests from orders such as Lepidoptera and Coleoptera [4,8,9]. Although phoxim is highly toxic to silkworms, it decomposes rapidly upon light exposure, leading to a relatively short residual activity. Therefore, with a recommended pre-harvest interval of 3 to 5 days, it is still frequently used for managing leaf-eating pests in mulberry fields [10,11]. In sericultural practice, a rotational pesticide application strategy is commonly employed, where mulberry plots are treated in separate sections, and harvesting occurs only after the pre-harvest interval. While this ensures a continuous supply of mulberry leaves for silkworm rearing, it may inadvertently lead to chronic low-level phoxim exposure due to residual compounds persisting on leaves even after the pre-harvest interval. However, the potential impact of this exposure on silkworms has long been overlooked.

Extensive research has been conducted on the toxic effects of phoxim on silkworms. However, most studies primarily focused on fifth-instar larvae, with limited investigation of chronic low-dose effects during earlier developmental stages. In fifth-instar larvae, a phoxim concentration of 0.05 μg/mL did not induce overt poisoning symptoms but led to alterations in the levels of certain essential elements in the hemolymph and caused significant DNA damage in cells [12,13]. At higher concentrations (0.375 and 0.750 μg/mL), notable upregulation of genes associated with detoxification in the midgut was observed [14]. Although 1 μg/mL phoxim exposure did not cause silkworm mortality, it induced sublethal toxic effects, including significant reductions in both body weight and cocoon weight, along with disruptions in glucose and lipid metabolism [15,16,17]. At 2.5 μg/mL, mortality occurred, accompanied by oxidative stress in the tissues [18]. When the concentration reached 4.0 μg/mL, mortality further increased, and severe tissue damage was observed in the brain, midgut, and silk glands. This was accompanied by reactive oxygen species (ROS) accumulation and significant reductions in both the cocooning rate and cocoon shell weight [4,19,20,21,22,23,24,25,26,27]. These studies demonstrate that short-term phoxim exposure adversely affects silkworm growth and development, with silk production significantly suppressed at high concentrations (4.0 μg/mL). Under real-world farming conditions, mulberry leaves may still contain low residual levels of phoxim even after the pre-harvest interval. Silkworms feeding on these leaves show no obvious toxic symptoms. However, it remains unclear whether long-term consumption could negatively affect the silk glands and lead to potential losses in silk production.

Silk proteins are the main constituents of silkworm cocoons, accounting for approximately 100% of their weight [28]. The silk gland is a vital organ in silkworms, responsible for synthesizing silk proteins and critical to determining silk yield and quality. The posterior silk gland (PSG), located in the posterior abdomen, serves as the primary site for fibroin synthesis [29,30]. As the main component of silk, fibroin consists of three subunits: the fibroin heavy chain (Fib-H-), which accounts for over 70% of fibroin, the fibroin light chain (Fib-L), and the P25 protein [31,32]. The expression regulation of *Fib-H*, *Fib-L*, and *P25* in silkworm is a critical determinant of silk quality and yield [31,33,34].

Fibroin synthesis is tightly regulated by various factors, particularly hormones [35,36,37]. Juvenile hormone (JH) and ecdysone play crucial roles in regulating fibroin expression [38]. The application of JH analogs during the early fifth-instar larval stage extends the development period, leading to both increased body weight and enhanced silk production [39]. Zhao et al. further demonstrated that JH regulated *Fib-H* transcription through the basic helix-loop-helix (bHLH) transcription factor *Dimm*. Specifically, dsRNA-mediated knockdown of *Kr-h1* mRNA significantly downregulated *Dimm* expression, resulting in reduced *Fib-H* levels [12]. Notably, ecdysone exhibits a dose-dependent effect: low doses of ecdysone can promote silk gland development and enhance its function, whereas high doses induce gland degeneration and metamorphosis [38]. Collectively, these studies highlight the crucial role of hormonal regulation in fibroin synthesis.

This study aims to investigate the potential impacts and biological mechanisms of chronic exposure to low doses of phoxim on silk production in silkworms, focusing on multiple perspectives including growth, histopathology, enzyme activity, and gene expression. The objective is to evaluate the safety of phoxim use in silkworm farming environments and provide a theoretical basis for the scientific and rational application of phoxim in pest control within mulberry fields.

## 2. Materials and Methods

### 2.1. Insect Strains and Chemicals

Larvae of *B. mori* (strain: Jingsong × Haoyue) were reared on fresh mulberry leaves at 25 ± 1 °C under a 12-h light/dark cycle. Phoxim (CAS: 14816-18-3, purity: 99%) was purchased from Aladdin (Shanghai, China).

### 2.2. Toxicity Assessment

Phoxim was dissolved in acetone to form a stock solution and then diluted with double-distilled water to obtain working concentrations of 0.550, 0.578, 0.606, 0.637, 0.669, 0.702, and 0.737 μg/mL. Fresh mulberry leaves were immersed in these solutions for 1 min, air-dried at room temperature, and provided to first-day second-instar larvae. Each concentration group contained 60 silkworms, divided into three replicates. Mortality rates were recorded at 96 h, and the LC_50_ (lethal concentration 50%) value with 95% confidence intervals was calculated according to the Spearman–Karber method [40].

### 2.3. Chronic Exposure and Sample Collection

Preliminary investigations showed that phoxim residues in mulberry leaves ranged from 5 to 15 μg/kg 5 days after application at the recommended dose. Based on the LC_50_ value (0.632 μg/mL) for second-instar larvae determined in this study and actual residue levels, four treatment groups were established: 1/50 LC_50_ (0.013 μg/mL), 1/20 LC_50_ (0.032 μg/mL), 1/8 LC_50_ (0.079 μg/mL), and 1/2 LC_50_ (0.316 μg/mL). Continuous exposure was achieved using the leaf-dipping method from the first day of the second instar until cocooning. Mulberry leaves were immersed in phoxim solutions for 1 min, air-dried at room temperature, and subsequently fed to larvae. Each treatment group contained 390 silkworms, divided into three replicates. Body weight was recorded at 48 h of each instar. On the third day of the fifth instar, larvae PSG were dissected on ice and stored at −80 °C for subsequent experiments. Each treatment group contained 30 silkworms, which were used to determine the cocoon shell ratio after pupation. The cocoon shell ratio was calculated as the percentage of the cocoon shell weight relative to the total cocoon weight.

### 2.4. Histopathological Analysis of Silk Glands

On the third day of the fifth instar, the larvae were dissected, and the PSG was carefully excised. The tissues were fixed in 4% paraformaldehyde for 24 h, dehydrated using a graded ethanol series, cleared in xylene, and embedded in paraffin. Sections of 5 μm thickness were cut, mounted onto glass slides, and dried at 37 °C overnight. Sections were routinely processed for histopathology and stained with H&E. The stained sections were observed and images were captured using a Nikon Eclipse C1 optical microscope equipped with camera (Nikon, Tokyo, Japan).

### 2.5. RNA Extraction and Quantitative Real-Time PCR (qRT-PCR)

Total RNA was isolated from the PSG using the RNA-easy Isolation Reagent (Vazyme Biotech, Nanjing, China) following the manufacturer’s protocol. RNA concentration and purity were determined by measuring the absorbance at 260 nm and 280 nm using a Nano-100 microspectrophotometer (Allsheng, Hangzhou, China). Reverse transcription of total RNA to cDNA was conducted using the Reverse Transcriptase Kit (Vazyme Biotech, Nanjing, China) as per the manufacturer’s instructions, which includes a step to eliminate residual genomic DNA. qRT-PCR was conducted on a LightCycler^®^ 96 Real-Time PCR system (Roche, Basel, Switzerland) using the PerfectStart^®^ Green qPCR SuperMix kit (TransGen Biotech, Beijing, China). No-RT control reactions were included to confirm the absence of genomic DNA contamination. Relative gene expression levels were calculated according to Livak and Schmittgen [41]. *RP49* was used as the internal reference gene. Primers for qRT-PCR were designed utilizing Primer 6.0 software (Supplemental Table S1).

### 2.6. Enzyme Activity Determination

Malondialdehyde (MDA) levels, peroxidase (POD) activity, and superoxide dismutase (SOD) activity were measured using commercial assay kits (Jiancheng, Nanjing, China) following the manufacturer’s protocols. Total protein levels were determined using the Bradford method.

### 2.7. Assay of JH and Ecdysone Titers

Hormone titers in the PSG were quantified utilizing insect JH and ecdysone enzyme-linked immunosorbent assay (ELISA) kits (Mlbio, Shanghai, China), according to the manufacturer’s instructions.

### 2.8. Statistical Analysis

All data were processed using SPSS software (version 27.0, IBM, Chicago, IL, USA). Normality was assessed with the Shapiro–Wilk test. For data meeting the normality assumption, homogeneity of variances was further examined using Levene’s test. Normally distributed data with equal variances were analyzed by one-way analysis of variance (ANOVA), followed by Dunnett’s post hoc test for comparisons against the control group [42]. Results were presented as mean ± standard error (SE). The cocoon shell ratio, which violated the normality assumption, was analyzed with the Kruskal–Wallis H test, and pairwise comparisons were performed using the Mann–Whitney *U* test [43]. Statistical significance was defined as *p* < 0.05.

## 3. Results

### 3.1. Acute Toxicity of Phoxim to Second-Instar Silkworms

Acute exposure resulted in dose-dependent phenotypic changes, including head nystagmus, chaotic crawling, and gastric juice vomiting. The 96-h LC_50_ for phoxim in second-instar larvae was calculated as 0.633 μg/mL, with a 95% confidence interval of 0.624 to 0.641 μg/mL. The toxicity regression equation is expressed as follows:

Y = −2.410767 + 0.00864 X (R^2^ = 0.99)

### 3.2. Effects on Growth and Cocooning of Silkworms

**Figure 1:**
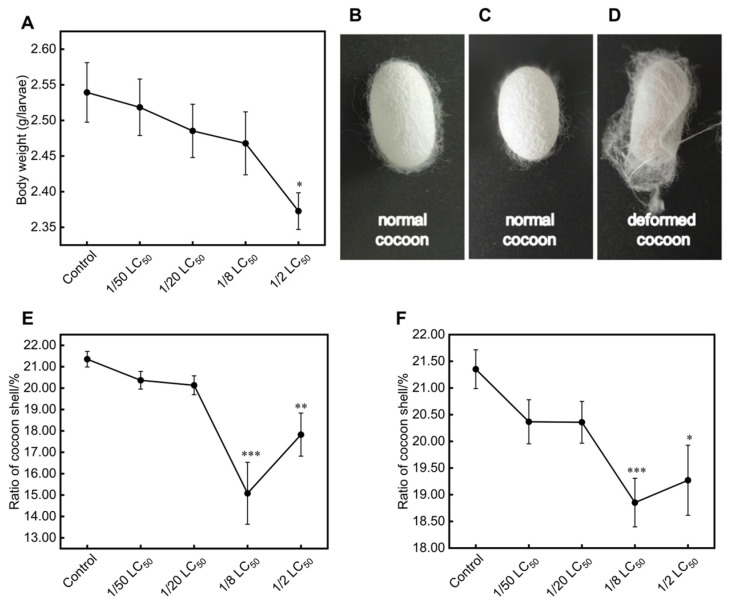
Effects of chronic low-dose phoxim exposure on the growth of silkworms. (**A**) Body weight of fifth-instar larvae; (**B**) Cocoon shape in the control group; (**C**) Normal cocoons shape in the phoxim-treated groups; (**D**) Deformed cocoons in the phoxim-treated groups; (**E**) Cocoon shell ratio of all cocoons; (**F**) Cocoon shell ratio excluding deformed cocoons (e.g., thin shells). Significant difference vs. Control (* *p* < 0.05, ** *p* < 0.01, *** *p* < 0.001).

Based on the LC_50_ results obtained above, a long-term exposure experiment was conducted using several concentrations of phoxim. Throughout the exposure period, no apparent signs of poisoning were observed in the treatment groups compared with the control group, either in body size or appearance. However, it is noteworthy that by the fifth instar, larvae in the 1/2 LC_50_ group exhibited a 7% reduction in body weight compared with the control group (*p* < 0.05, Figure 1A). The body weights of the 1/50 LC_50_, 1/20 LC_50_, and 1/8 LC_50_ groups decreased by 1%, 2%, and 3%, respectively, but these reductions were not statistically significant compared with the control group (Figure 1A). The larval period duration showed no significant differences among the groups (*p* > 0.05), but the cocooning duration was slightly prolonged in the treatment groups. The cocoons in the control group were uniform and symmetrical (Figure 1B), whereas some cocoons in the phoxim-treated groups exhibited irregular shapes and thinner shells (Figure 1D). The cocoon shell ratio decreased in the 1/50 LC_50_ and 1/20 LC_50_ groups compared with the control group, but there was no significant difference. However, the 1/8 LC_50_ and 1/2 LC_50_ groups exhibited a significant reduction in the cocoon shell ratio (*p* < 0.01, Figure 1E). Even after excluding defective cocoons, including thin-shelled ones, cocoon weight remained significantly lower in the 1/8 LC_50_ and 1/2 LC_50_ groups compared with the control group (Figure 1F).

### 3.3. Effects on Silk Gland Damage and Oxidative Stress

**Figure 2:**
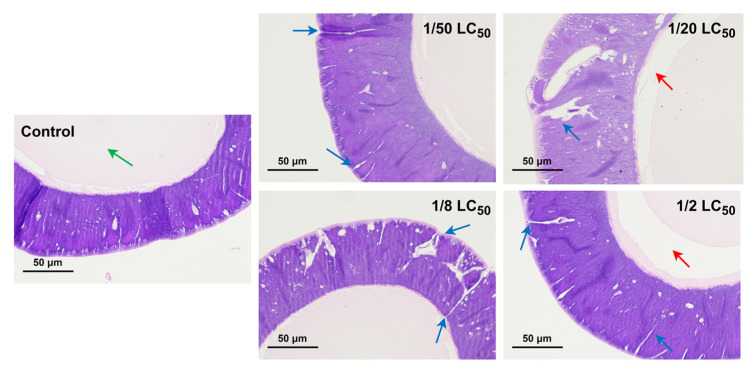
Histopathological analysis of the PSG in fifth instar silkworm exposed to phoxim. Green arrows indicate protein aggregates; blue arrows indicate damage to the glandular membranes; red arrows indicate vacuolization in the gland lumen. Scale bar = 50 μm.

Histopathological results showed that the control group had normal structure, with fully filled lumen and no significant pathological changes (Figure 2). In contrast, glandular membrane damage was observed in the 1/50 LC_50_ group, and the 1/20 LC_50_ and 1/8 LC_50_ groups showed sparse epithelial cells and severe glandular membrane damage (Figure 2). Although no obvious damage to the glandular membrane was observed in the 1/2 LC_50_ group, vacuolization within the gland lumen was more pronounced (Figure 2).

**Figure 3:**
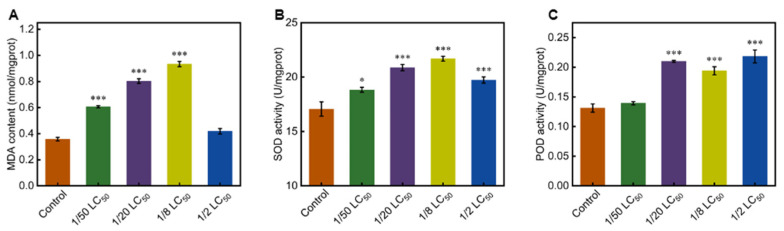
Oxidative responses in the PSG of silkworms exposed to phoxim. (**A**) Content of malondialdehyde (MDA); (**B**) Superoxide dismutase (SOD) activity; (**C**) Peroxidase (POD) activity. Significant difference vs. control (* *p* < 0.05, *** *p* < 0.001).

Given the histopathological evidence of silk gland damage, we hypothesized that oxidative stress might underlie these morphological alterations. The MDA content in the PSG was significantly increased in the 1/50 LC_50_, 1/20 LC_50_, and 1/8 LC_50_ groups compared with the control group, while the increase in the 1/2 LC_50_ group was not statistically significant (Figure 3A). SOD activity was significantly higher in all treatment groups (Figure 3B), and POD activity showed significant increases in the 1/20 LC_50_, 1/8 LC_50_, and 1/2 LC_50_ groups (Figure 3C). These results demonstrate that phoxim exposure caused significant alterations in multiple oxidative stress-related parameters in the PSG.

### 3.4. Effects on Fibroin Synthesis Gene Expression

**Figure 4:**
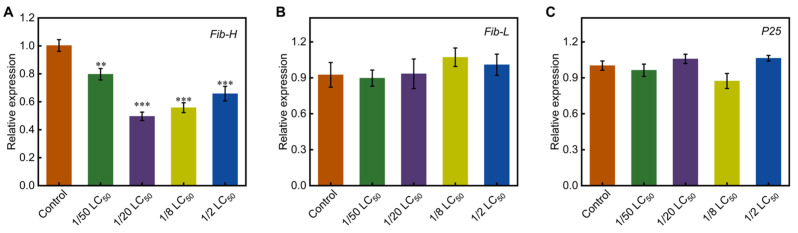
Effects of chronic low-dose phoxim exposure on the transcript levels of fibroin protein synthesis genes. (**A**) Relative expression level of *Fib-H*; (**B**) Relative expression level of *Fib-L*; (**C**) Relative expression level of *P25*. Significant difference vs. control (** *p* < 0.01, *** *p* < 0.001).

Fibroin is primarily composed of three components: Fib-H, Fib-L, and P25. To evaluate the impact of phoxim on fibroin synthesis, the transcription levels of fibroin genes in the PSG were measured. The results showed that *Fib-H* expression was significantly down-regulated, with levels decreasing to 0.80- (S1), 0.50- (S2), 0.56- (S3), and 0.66-fold (S4) of the control group, respectively. However, the transcription levels of the *Fib-L* and *P25* genes did not show significant differences compared with the control group (Figure 4).

### 3.5. Effects on the Titer of JH and Ecdysone

**Figure 5:**
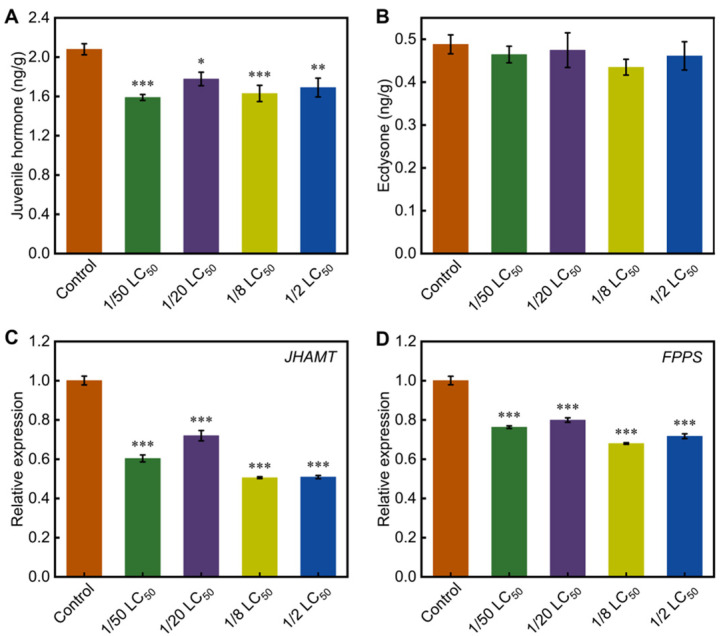
Effects of phoxim on hormone titers and related gene expression in the silk glands. (**A**) Juvenile hormone (JH) titer; (**B**) Ecdysone titer; (**C**) Relative expression level of *JHAMT*; (**D**) Relative expression level of *FPPS*. Significant difference vs. control (* *p* < 0.05, ** *p* < 0.01, *** *p* < 0.001).

Given the role of hormonal regulation in fibroin synthesis, we quantified JH and ecdysone titers in the PSG. The JH titer was significantly lower in the treatment groups compared with the control group (Figure 5A). However, ecdysone titer remained unchanged (Figure 5B). Consistently, it was found that the transcription levels of *Juvenile Hormone Acid O-Methyltransferase* (*JHAMT*) and *Farnesyl Pyrophosphate Synthase* (*FPPS*) were significantly downregulated in the treatment groups (Figure 5C,D). These findings suggest that phoxim disrupts hormonal homeostasis in the silk gland.

### 3.6. Effects on JH Signaling Pathway Genes Related to Silk Protein Synthesis

**Figure 6:**
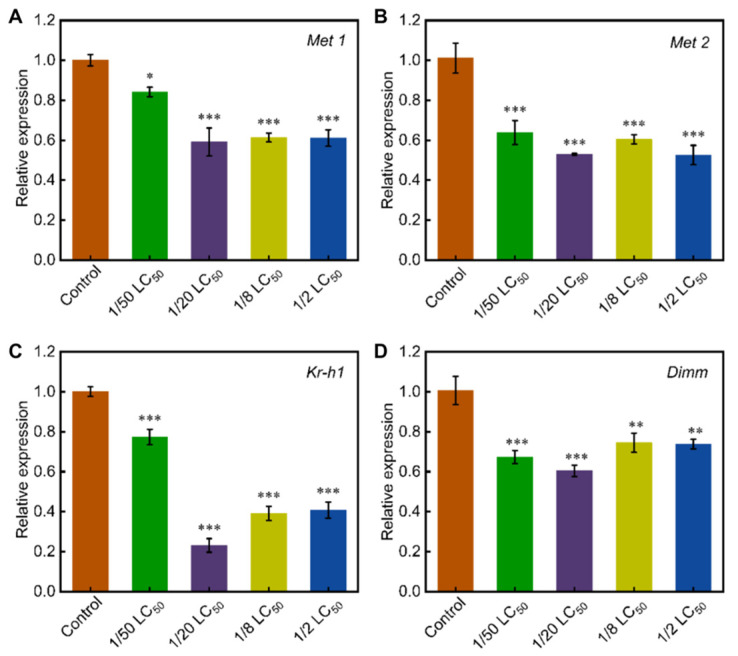
Effects of chronic low-dose phoxim exposure on the transcript levels of genes involved in the JH signaling pathway. (**A**) Relative expression level of *Met1*; (**B**) Relative expression level of *Met2*; (**C**) Relative expression level of *Kr-h1*; (**D**) Relative expression level of *Dimm*. Significant difference vs. control (* *p* < 0.05, ** *p* < 0.01, *** *p* < 0.001).

To investigate the molecular regulatory mechanisms, we measured the transcription levels of genes related to *Fib-H* within the JH signaling pathway. *Met1* and *Met2* function as bHLH-PAS transcription factors that act as juvenile hormone (JH) receptors [44]. All phoxim treatment groups showed significant downregulation of *Met1* and *Met2* transcription. Specifically, *Met1* expression was reduced to 0.84-, 0.59-, 0.61-, and 0.61-fold the control levels, while *Met2* expression was reduced to 0.64-, 0.53-, 0.60-, and 0.53-fold the control (Figure 6A,B). Similarly, *Kr-h1*, a downstream mediator of JH signaling [45], was significantly downregulated, with transcript levels reduced to 0.77-, 0.23-, 0.39-, and 0.41-fold of the control group, respectively (Figure 6C). *Dimm* is specifically expressed in silk glands and directly regulates the expression of *Fib-H* via the JH–Met–Kr-h1 pathway [12]. The transcript level of *Dimm* was also significantly downregulated in the treatment groups, with levels reduced to 0.67-, 0.60-, 0.75-, and 0.74-fold of the control group, respectively (Figure 6D).

## 4. Discussion

Phoxim exposure has been shown to impair silkworm growth and reduce economic performance. Li observed that third-day fifth-instar silkworms exposed to 1 μg/mL phoxim for 96 h exhibited mild poisoning symptoms, such as body wrinkling and rapid, uncontrollable head movements. Additionally, this treatment significantly reduced both body weight and cocoon weight [17]. A higher concentration (4 μg/mL) caused severe vomiting, behavioral abnormalities, and declines in cocooning parameters after 36 h of exposure [5,19]. In this study, we found that chronic low-dose phoxim exposure starting from the second instar did not result in obvious acute poisoning symptoms. However, by the fifth instar, silkworms in the treatment groups exhibited reduced body weight compared with the control group, with a particularly significant reduction observed in the 1/2 LC_50_ group (Figure 1A). This indicates that chronic low-dose phoxim exposure suppresses silkworm growth despite the absence of acute toxicity. Notably, decreased cocoon shell ratios were observed in the treatment groups (Figure 1E,F), especially in the 1/8 LC_50_ and 1/2 LC_50_ groups. It is worth noting that deformed cocoon formation occurred in the treatment groups (Figure 1D). These findings suggest that the effects of chronic low-dose phoxim exposure on silkworms are latent and cumulative, different from the immediate toxicity caused by acute high-dose exposure. Our study highlights that such chronic phoxim exposure may impair silk production, potentially reducing sericulture profitability. These results underscore the need to reassess phoxim safety standards in sericulture and highlight the importance of stricter pesticide residue limits to mitigate economic risks.

It has been reported that short-term exposure to high doses of phoxim can affect the silk glands of silkworms. For instance, Li et al. reported that treating third-day fifth-instar larvae with 4 μg/mL phoxim for 36 h resulted in ruptured gland membranes and significant vacuolation within the gland lumen [5,19,20]. Similarly, Cheng et al. orally administered 5 μg phoxim to third-day fifth-instar larvae and observed morphological damage in the silk gland 48 h later, characterized by the presence of fissures and vacuoles [46]. In this study, we found that long-term exposure to even low doses of phoxim caused varying degrees of tissue damage (Figure 2). The 1/2 LC_50_ group exhibited significant vacuolation within the gland lumen, while the 1/8 LC_50_ and 1/20 LC_50_ groups showed membrane ruptures, with less pronounced vacuolation. The S1 treatment group displayed only minor damage, mainly characterized by slight fissures in the gland membrane (Figure 2). The damage in these groups was not as severe as that caused by high-dose, short-term exposure. However, the cumulative effects should not be underestimated, as this latent damage may progressively worsen under chronic exposure conditions.

In this study, oxidative stress responses were detected in the silkworms following phoxim exposure. Compared with the control group, MDA content was significantly elevated in all treatment groups except 1/2 LC_50_ (Figure 3A). This suggests that chronic exposure to low doses of phoxim can induce lipid peroxidation in the silk glands of silkworms. Our results are similar to those of Li et al., who reported a significant increase in MDA content in the silk glands of fifth-instar silkworms following 36-h treatment with 4 μg/mL phoxim [20]. Additionally, Yu et al. observed elevated MDA levels in the fat body and midgut of fifth-instar silkworms exposed to 2.5 μg/mL phoxim, with further increases occurring with extended exposure [47]. While previous studies primarily focused on the effects of short-term exposure to sub-lethal doses, our research highlights the potential risks of chronic low-dose phoxim exposure, which lead to prolonged oxidative stress and subsequent tissue damage in silkworms. High MDA levels indicate tissue and cell membrane damage [48]. Although the 1/2 LC_50_ group showed no significant increase in MDA content, the recorded pathological changes suggest that this exposure may induce cellular damage through alternative mechanisms. This reveals that even in the absence of acute toxicity symptoms, chronic low-dose exposure to phoxim may lead to the accumulation of lipid peroxidation products within the silk glands of silkworms, thereby heightening the risk of tissue damage [49].

The activities of SOD and POD in the silk glands were elevated in all treatment groups (Figure 3B,C). This suggests that the antioxidant system was activated in response to oxidative stress induced by phoxim exposure. Antioxidant enzymes function by scavenging excess free radicals and protecting cells from damage [50]. The elevation in antioxidant enzyme activity is a strategy to counteract oxidative stress and minimize damage from ROS [51]. Contrary to our findings showing elevated SOD activity, Li et al. reported significantly suppressed SOD activity in the silk glands of silkworms exposed to 4 μg/mL phoxim [20]. This discrepancy may be due to the different phoxim concentrations used in the two studies. High-dose exposure likely impaired antioxidant defenses, whereas our chronic low-dose exposure activated the antioxidant system to mitigate oxidative stress.

Silk fibroin is a primary protein constituting the silk produced by silkworms, and its synthesis genes (such as *Fib-H* and *Fib-L*) are strictly regulated at the transcriptional level [19,52]. It has been demonstrated that phoxim exposure disrupts the normal synthesis of silk fibroin in silkworms, significantly reducing silk production [25]. For instance, when 3-day-old fifth-instar larvae were fed mulberry leaves treated with 4 μg/mL phoxim, the transcription levels of *Fib-H*, *Fib-L*, and *P25* were significantly downregulated [19,20]. In this study, the transcription level of *Fib-H* was downregulated in the treatment groups (Figure 4A), while the transcription levels of *Fib-L* and *P25* did not show significant changes (Figure 4B,C). This indicates selective inhibition under chronic low-dose exposure. Similar specificity was reported in pyriproxyfen-treated silkworms, where *Fib-H* was suppressed [53]. Notably, despite the downregulation of *Fib-H*, the 1/50 LC_50_ and 1/20 LC_50_ groups maintained near-normal cocoon shell ratios (Figure 1C) likely due to prolonged silk-spinning periods compensating for reduced synthesis efficiency. In contrast, the 1/8 LC_50_ and 1/2 LC_50_ groups exhibited severe glandular vacuolization (Figure 2), which disrupted glandular function and resulted in deformed cocoons with significantly reduced shell ratios.

JH and ecdysone are critical hormones in insects, and their titers and ratios directly influence insect growth and development, particularly the synthesis of silk fibroin [30,54,55]. In this study, chronic low-dose phoxim exposure significantly reduced JH titers in the silk glands (Figure 5A,B), while ecdysone titers remained unaltered. Further analysis revealed that the transcription levels of genes involved in JH synthesis, such as *FPPS* and *JHAMT*—encoding key enzymes involved in the early and critical steps of JH synthesis—were significantly downregulated in the treatment groups (Figure 5C,D). This indicates a severe disruption of the JH biosynthesis pathway [56]. The JH signaling pathway plays a crucial role in regulating the expression of genes encoding silk fibroin [12]. Specifically, JH regulates *Dimm* expression through the JH–Met–Kr-h1 signaling pathway, which in turn promotes the expression of *Fib-H* in the silk glands [12,36,57]. In silkworm, Met1 and Met2 serve as JH receptors, with Met1 playing a central role in regulating *Kr-h1*. Our data showed significant downregulation of *Met1*, *Met2*, and *Kr-h1* transcription in the treatment groups (Figure 6A,B), which suggests suppression of the JH signaling pathway. Meanwhile, the expression of *Dimm* was also reduced, which lead to reduced *Fib-H* expression. Consequently, these changes resulted in the observed reduction in cocoon yield.

## 5. Conclusions

Phoxim is widely used to control mulberry pests and is generally considered safe for silkworms due to its rapid photodegradation and low residue levels after the pre-harvest interval. However, the potential impact of chronic low-dose exposure on silkworms, particularly regarding silk production, has long been overlooked. This study addresses this gap by demonstrating that chronic exposure to phoxim, even at environmentally relevant levels, causes silk gland damage, reduces JH titers, and impairs silk production. Notably, we identified a previously unreported mechanism in which phoxim suppresses the expression of the fibroin gene *Fib-H* via the JH–Met–Kr-h1–Dimm signaling pathway. These findings offer important molecular insights into how pesticide residues compromise silk biosynthesis and underscore the necessity of reassessing pesticide safety thresholds in sericulture.

## Data Availability

The data presented in this study are available upon request from the corresponding author.

## Supplementary Materials

The following supporting information can be downloaded at: https://www.mdpi.com/article/10.3390/toxics13060427/s1, Table S1: Primer sequences used in this study.
