# Supplementary material for: Chronic Low-Dose Phoxim Exposure Impairs Silk Production in Bombyx mori L. (Lepidoptera: Bombycidae) by Disrupting Juvenile Hormone Signaling-Mediated Fibroin Synthesis
Source: Toxics. 2025 May 23;13(6):427. doi: 10.3390/toxics13060427 (PMC12196826; doi:10.3390/toxics13060427)
Supplement: Supplementary file 1 [file toxics-13-00427-s001.zip › toxics-3609849-supplementary.pdf]

Table S1. Primer sequences used in this study.

Gene name	Accession number	Forward primers (5′-3′)	Reverse primers (5′-3′)
RP49	NM_001098282.2	GCATCAATCGGATCGCTATG	GGACCTTACGGAATCCATTTG
Fib-H	NM_001113262.1	ACAAGGTGCAGGAAGTGC	AGCAATTCACACAAGGCAGT
Fib-L	NM_001044023.1	CCGGAGGTGGAAGAATCTAT	GGTTATGTAGGCAGCGATGT
P25	NM_001145941.1	CCCTGCTACTTGGACGATT	GATTATGGTCGACGTAGGTG
JHAMT	NM_001043436.1	AAATAGGCAGGGCGGTGGTA	AAAGCAGCTGGCTCTCACTA
FPPS	NM_001043424.1	ACTAGCGTCACCAATTCTGATGCC	GCAGCTTCAGCAGTCTCTTCGG
Met2	NM_001114985.1	CGTGGCGAAGCTCCTGAATGC	GGTGACCAACGAGATACGCTGTTC
Kr-h1	NM_001177861.1	TACAACACCAATGGCTGCCGATG	ATGGCGGTGAACTAGGTGGAGTAG
Dimm	NM_001294284.1	TCAACCGAGCTTTTGAGGAT	AATACAAATGCGGGTTCCAC
